# Azithromycin Inhibits Biofilm Formation by *Staphylococcus xylosus* and Affects Histidine Biosynthesis Pathway

**DOI:** 10.3389/fphar.2018.00740

**Published:** 2018-07-10

**Authors:** Wenya Ding, Yonghui Zhou, Qianwei Qu, Wenqiang Cui, Bello Onaghise God’spower, Yanyan Liu, Xueying Chen, Mo Chen, Yanbei Yang, Yanhua Li

**Affiliations:** ^1^Department of Veterinary Medicine, Northeast Agricultural University, Harbin, China; ^2^Heilongjiang Key Laboratory for Animal Disease Control and Pharmaceutical Development, Harbin, China

**Keywords:** *Staphylococcus xylosus*, biofilms, azithromycin, ribosomal protein, histidine biosynthesis pathway, imidazole glycerophosphate dehydratase

## Abstract

*Staphylococcus xylosus*, a coagulase-negative, non-pathogenic bacterium, responsible for opportunistic infections in humans and bovine mastitis, has the ability to form biofilms, which are responsible for persistent infections and antibiotic resistance. In our study, azithromycin significantly inhibited biofilm formation by altering protein expression. Of the 1764 proteins measured by the isobaric Tag for Relative and Absolute Quantification (iTRAQ) technique, only 148 proteins showed significantly different expression between the azithromycin-treated and untreated cells. Most ribosomal proteins were markedly up-regulated, and the expression of the proteins involved in histidine biosynthesis, which, in turn, influence biofilm formation, was down-regulated, particularly imidazole glycerophosphate dehydratase (IGPD). Previously, we had observed that IGPD plays an important role in biofilm formation by *S. xylosus*. Therefore, *hisB* expression was studied by real-time PCR, and the interactions between azithromycin and IGPD were predicted by molecular docking analysis. *hisB* was found to be significantly down-regulated, and six bond interactions were observed between azithromycin and IGPD. Many active atoms of azithromycin did not interact with the biologically active site of IGPD. Surface plasmon resonance analysis used to further study the relationship between IGPD and azithromycin showed minimum interaction between them. Histidine content in the azithromycin-treated and untreated groups was determined. We noted a slight difference, which was not consistent with the expression of the proteins involved in histidine biosynthesis. Therefore, histidine degradation into glutamate was also studied, and we found that all proteins were down-regulated. This could be the reason why histidine content showed little change between the treated and untreated groups. In summary, we found that azithromycin is a potential inhibitor of *S. xylosus* biofilm formation, and the underlying mechanism was preliminarily elucidated in this study.

## Introduction

*Staphylococcus xylosus* is a very important opportunistic pathogen that causes infections in humans and chronic mastitis in bovines (Król et al., 2016; Bochniarz et al., 2017). Its ability to form biofilms deems it notorious for persistent infections, antibiotic resistance, and evasion of immune response (Azelmad et al., 2017; Kumar et al., 2017). Therefore, biofilm-associated infections are considered a major problem in modern medicine, affecting millions worldwide (Oliveira et al., 2017).

Biofilms are formed by microbial cells that adhere to each other, within a matrix of extracellular polymeric substance (Jamal et al., 2017). Biofilm formation is involved in several complex molecular mechanisms such as metabolism of nitrogen and carbon and biosynthesis of amino acids, especially L-histidine, which is implicated in biofilm formation, as shown by preliminary studies (Cabral et al., 2011; Xu et al., 2017). Imidazole glycerophosphate dehydratase (IGPD) is an important enzyme in the L-histidine synthesis pathway, and it can catalyze the dehydration of imidazole glycerol phosphate (IGP) to imidazole acetol phosphate (IAP). Our previous study showed that IGPD plays a key role in biofilm formation by *S. xylosus* (Zhou et al., 2018).

Azithromycin, a macrolide antibiotic, is derived from erythromycin. It can achieve high intracellular concentrations, reduce acute inflammation, and help to resolve chronic inflammation by promoting long-term repair and healing (Casasmaldonado, 2017). Azithromycin is recommended as first-line therapy for bacterial infections in American and European guidelines (Descours et al., 2017). It is a broad-spectrum antibiotic useful against many gram-positive and gram-negative bacteria such as *Streptococcus pneumoniae, Haemophilus influenzae*, and *Chlamydophila pneumoniae* (Wang et al., 2018). Azithromycin can significantly inhibit biofilm formation by *Pseudomonas aeruginosa* by reducing swarming and twitching motility (Bahari et al., 2017). It can decrease α-hemolysin production and biofilm formation by methicillin-resistant *Staphylococcus aureus* (Gui et al., 2014). Azithromycin can also interfere in biofilm formation by *Porphyromonas gingivalis, H. influenzae*, and *Candida albicans* (Gui et al., 2014; Washington et al., 2015; Yang et al., 2016; Bahari et al., 2017). However, there are no studies about azithromycin-induced inhibition of *S. xylosus* biofilm formation and the underlying mechanism.

**SCHEME 1:**
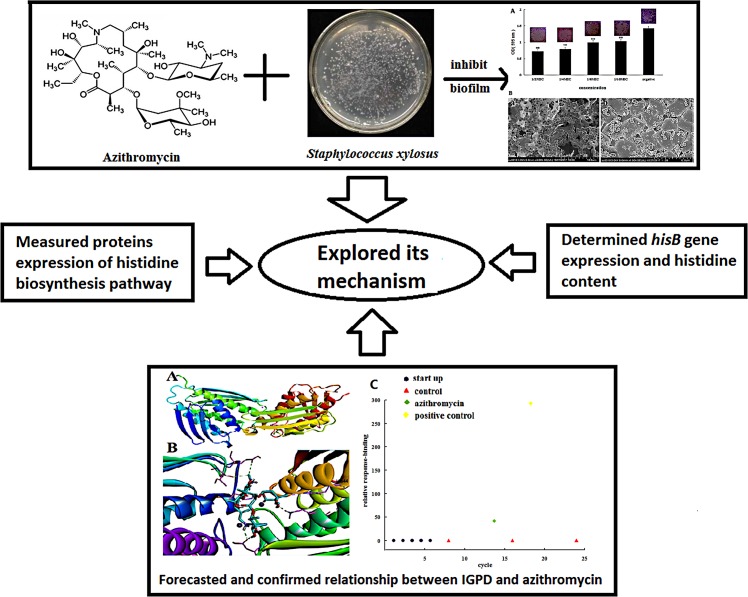
Azithromycin inhibited the formation of biofilms by *Staphylococcus xylosus* 700404; the underlying mechanism was explored.

*Staphylococcus xylosus* strains can form biofilms, which can cause persistent, slowly progressing, chronic infections (Rasamiravaka et al., 2015). Thus, inhibiting biofilm formation might be an important strategy for eradicating persistent bacterial infections. Recent studies have shown that azithromycin at the sub-minimal inhibitory concentration (sub-MIC) inhibits biofilm formation (Washington et al., 2015; Das et al., 2016). Our preliminary experiments showed that sub-MIC azithromycin could decrease biofilm formation by *Streptococcus suis* (Yang et al., 2016). However, the relationship between azithromycin and biofilm formation by *S. xylosus* remains poorly understood. In this study, we aimed to identify a drug that could inhibit biofilm formation by *S. xylosus* as well as elucidate the underlying mechanism. To provide relatively comprehensive understanding of its mechanism, we investigated the following: (1) the differential proteins present in cells treated and untreated with azithromycin; (2) the modifications to *hisB* expression caused by azithromycin; and (3) the interaction between azithromycin and IGPD (**Scheme 1**). Our research will explore the anti-biofilm formation potential of azithromycin inhibit *S. xylosus* and lay the foundation to further develop the use of azithromycin.

## Materials and Methods

### Bacterial Growth Conditions and Determination of MIC

*Staphylococcus xylosus* ATCC 700404 was used in this study. It was grown overnight in Tryptic Soy Broth (TSB, Oxoid) at 37°C with constant shaking. Azithromycin MIC was determined by microbroth dilution assay, as described in the Clinical and Laboratory Standards Institute guidelines. Control (with TSB alone) and negative control (with bacteria alone) were included, and the experiments were performed in triplicate.

### Biofilm Formation Inhibition by Azithromycin

#### Tissue Culture Plate (TCP) Assay

Tissue culture plate assay was used to test the inhibitory effect of azithromycin on biofilm formation (Chen et al., 2017). A culture suspension of *S. xylosus* 700404 at the mid-exponential phase of growth was diluted with TSB to an optical density of 0.1 at 595 nm (OD_595_). Next, 100 μL of this suspension and 100 μL of azithromycin were added to each well of a 96-well microplate, at final azithromycin concentrations of 1/2-MIC (0.25 μg/mL), 1/4-MIC (0.125 μg/mL), 1/8-MIC (0.0625 μg/mL), and 1/16-MIC (0.03125 μg/mL), respectively. In addition, control (with TSB alone) and negative control (with bacteria alone) were included after incubation at 37°C for 24 h without shaking. The supernatant was removed, the wells were rinsed three times with phosphate-buffered saline (PBS; pH 7.2), 200 μL of 99% methanol was added to the wells to fix the biofilms, and then, the plates were emptied after 15 min and stained for 5 min with 200 μL of 2% crystal violet per well (Ding et al., 2017). The wells were rinsed with PBS (pH 7.2), and the dye was resolubilized with 200 μL of 33% (v/v) glacial acetic acid per well (Ding et al., 2017). All wells were then measured using a Tecan GENios Plus microplate reader (Tecan, Austria) at 595 nm (Ding et al., 2017). The experiments were performed in triplicate.

#### Scanning Electron Microscopy (SEM)

Scanning electron microscopy was performed according to a previously described procedure (Ding et al., 2017). Briefly, a mid-exponential culture suspension of *S. xylosus* 700404 and 1/2-MIC (0.25 μg/mL) of azithromycin were added to a 6-well microplate (Corning Co., Ltd., United States), in which each well contained an 11-mm- × -11-mm sterilized rough organic membrane (Mosutech Co., Ltd., Shanghai, China) at the bottom. After incubation at 37°C for 24 h without shaking, the organic membranes were taken out and rinsed with PBS (pH 7.2). Then, the samples were fixed with 4% (w/v) glutaraldehyde for 6 h, and fixed to a black transparency by using 2% (w/v) osmium tetroxide (Ding et al., 2017). After dehydration, the samples were sputtered with gold and measured by SEM (FEI Quanta, Netherlands).

### iTRAQ Analysis

#### Protein Extract Preparation and iTRAQ Labeling

For biofilm formation, *S. xylosus* 700404 and *S. xylosus* supplemented with 1/2-MIC (0.25 μg/mL) azithromycin were grown in TSB at 37°C for 24 h. The biofilms were scraped and sonicated for 5 min (Bransonic 220; Branson Consolidated Ultrasonic Pvt. Ltd., Australia). The suspended samples were centrifuged at 4°C for 10 min (Ding et al., 2017). Protein samples were stored at -80°C for further analysis.

iTRAQ labeling was performed as previously described (Ding et al., 2017). The proteins were mixed with dithiothreitol, boiled for 5 min, ultra-filtered (Microcon-10 kD), resuspended in 100 mL of iodoacetamide (IAA; 50 mM IAA in urea) for 30 min in darkness, collected by centrifugation, and digested with trypsin (Promega) at 37°C. The peptides were obtained by filtration. Then, 30 mg of the peptides obtained from each sample were tagged according to the manufacturer’s recommendations (Applied Biosystems). The peptides were labeled as (Sample1)-117 and (Sample2)-118. After labeling, the iTRAQ-labeled peptide mixtures were pooled and separated by strong cationic exchange (SCX) chromatography by using a PolySULFOETHYL column (4.6 mm × 100 mm, 5 mm, 200 Å; PolyLC Inc., Columbia, MD, United States). The separation was run at a flow rate of 1 mL/min, by using a step gradient of phase B [500 mM KCl, 25% (v/v)] as follows: 0–10% for 2 min, 10–20% for 25 min, 45–100% for 5 min, and 100% for 8 min.

#### Liquid Chromatography (LC)-Mass Spectrometry (MS)/MS Analysis

iTRAQ-labeled samples were detected as described previously (Ding et al., 2017). Q Exactive mass spectrometer (Thermo Finnigan) fixed to EasynLC (Proxeon Biosystems, now Thermo Fisher Scientific) was used to test sample fractions. The iTRAQ-labeled peptide mixtures were injected and loaded onto a C18-reverse-phase column (100 mm × 75 mm; 3 mm) at a flow rate of 250 nL/min. The peptides were then separated by using a linear gradient of buffer B (80% acetonitrile and 0.1% formic acid) controlled by IntelliFlow technology over 140 min. MS analysis was performed at a resolution of 70,000 for 120 min with the resolution for higher-energy collisional dissociation (HCD) spectra set at 17,500 at m/z. The MS/MS spectra were searched using MASCOT engine (Matrix Science, London, United Kingdom; version 2.2) embedded into Proteome Discoverer 1.3 (Thermo Electron, San Jose, CA, United States) against the UniProt database and decoy database [peptide mass tolerance = 20 ppm, MS/MS tolerance = 0.1 Da, enzyme = trypsin, missed cleavage = 2, fixed modification: carbamidomethyl (C), iTRAQ8plex (K), iTRAQ8plex (N-term), variable modification: oxidation (M), FDR ≤ 0.01].

### Histidine Content Determination

Overnight cultured cells of *S. xylosus* 700404 were diluted with TSB (corresponding to 1 × 10^5^ colony-forming units/mL), and treated with 1/2-MIC (0.25 μg/mL) azithromycin; this mixture was incubated at 37°C for 24 h. Untreated *S. xylosus* 700404 served as the control. The assay was conducted using a previously described protocol (Macpherson, 1946). The cells were sonicated for 5 min (Bransonic 220; Branson Consolidated Ultrasonic Pvt. Ltd., Australia), and Pauly A (0.9% amino-sulfonic acid, 0.9% hydrochloric acid, and 5% sodium nitrite), cooled to 0°C, was mixed with the suspension and incubated for 5 min. Then, 4% sodium hydroxide was added, and the absorbance of samples was read at 476 nm by using a UV spectrophotometer (Shimadzu, Ltd., Japan).

### Real-Time PCR Analysis

Proteomic analysis showed IGPD (*hisB*) and formimidoylglutamase (*hutG*) to have most differential expression (*P* < 0.05), and therefore, these two proteins were selected for mRNA expression analysis. The 16sRNA gene was used as the internal gene. The primers used are listed in **Table 4**. Real-time PCR was performed as described in our previous study (Yang et al., 2016). *S. xylosus* culture (mid-log phase) was supplemented with 1/2-MIC (0.25 μg/mL) azithromycin and incubated at 37°C for 24 h, and untreated cells served as the control. The supplemented solution was centrifuged at 10,000 ×*g* for 5 min and treated with an RNASE REMOVER I (Huayueyang Ltd., Beijing, China). E.Z.N.A^TM^ bacterial RNA isolation kit was used to determine the total RNA levels. The relative expression was calculated using the 2^-ΔΔC_T_^ method.

### Molecular Docking Between IGPD and Azithromycin

Our previous study showed that IGPD could affect biofilm formation by *S. xylosus* 700404 (Chen et al., 2017; Zhou et al., 2018). Therefore, we investigated the interaction between azithromycin and IGPD by molecular docking. The 3D structure of IGPD was constructed by homology modeling technique and evaluated using Ramachandran Plot, Profile-3D analysis, and Qualitative Model Energy Analysis in our preliminary study (**Figure 3**) (Chen et al., 2017). Azithromycin was used to prepare ligands to generate 3D conformations. IGPD active site was predicted and the radius was set to 15 Å, and then, the docking study was performed according to the Schrodinger-Glide protocol under default conditions.

### Surface Plasmon Resonance (SPR) Analysis

Surface plasmon resonance method was used to further investigate the relationship between IGPD and azithromycin. The sensor surfaces were set up using the Biacore T200 system (GE Healthcare). EDC-NHS (70 μL) was used to activate the surface (CM7 chip; GE Healthcare) at a flow rate of 0.5 mL/min, IGPD was diluted to 30 μg/mL in 10 mM sodium acetate and immobilized in a flow cell on a sensor chip. In addition, 1 M ethanolamine was used to block the surface-activated groups after immobilizing IGPD. The running buffer used for immobilization contained 900 mL of 1.1× PBS and 100 mL of 100% methanol (pH 7.4). Azithromycin (320 nM) was injected (100 μL) at a flow rate of 2 mL/min. In addition, positive control (with ceftiofur) and control (with the mobile phase) were included. All experiments were performed at 25°C.

### Statistical Analysis

The values were calculated as the mean of individual experiments performed in triplicate, and compared with those of the control groups. The results were expressed as the mean + standard deviation (SD). *P* ≤ 0.05 indicated significant difference. Statistical comparison of the differences in biofilm formation, iTRAQ analysis, and histidine content was performed using the Wilcoxon test (SPSS 11.0.0 statistical software). Real-time PCR data were analyzed using repeated measurements in the -ΔCt model.

## Results

### Inhibition of Biofilm Formation by Azithromycin

**FIGURE 1:**
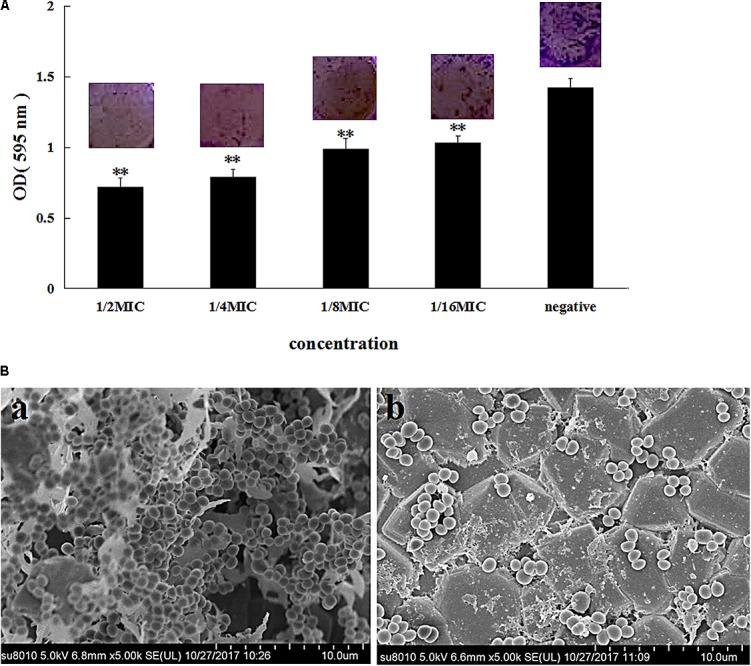
Effect of azithromycin on biofilm formation by *Staphylococcus xylosus* ATCC 700404. **(A)** Determination of biofilm formation by TCP. **(B)** SEM images of biofilm formation by cells untreated **(a)** or treated with azithromycin (1/2-MIC; **b**). Data are expressed as the mean + standard deviation (SD). A significant decrease (^∗∗^*P* < 0.05) was observed, compared to the control (*in vitro* biofilm formation).

The MIC of azithromycin against *S. xylosus* ATCC 700404 was found to be 0.5 μg/mL. **Figure 1A** showed that azithromycin can inhibit biofilm formation. With increasing concentrations of azithromycin in TCP assay, the OD values and violet clumps reduced, as well as biofilm formation by *S. xylosus* 700404 (**Figure 1A**). In **Figure 1B-a**, many clump-like structures could be seen in the control group, which indicate biofilm formation (Ding et al., 2017). Clump-like structures was barely observed in case of the 1/2-MIC-azithromycin-treated group (**Figure 1B-b**), indicating that azithromycin could significantly inhibit *S. xylosus* biofilm formation. The results of SEM are consistent with those of the TCP assay.

### Protein Expression Analysis

In this study, iTRAQ technology was utilized to better understand the significant differences proteins of 1/2-MIC-azithromycin-treated and untreated cells. About 1,764 proteins were detected, of which 148 were differentially expressed in *S. xylosus* cells treated with 1/2-MIC azithromycin (**Supplementary Table S1**). The distinct proteins had a fold-change ratio of >1.2 or <0.8 (*P* ≤ 0.05).

**FIGURE 2:**
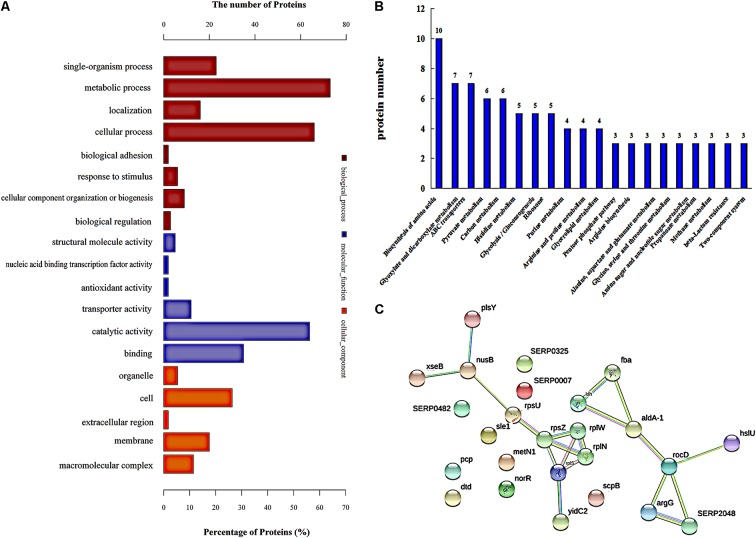
Protein expression analysis. **(A)** Functional annotation of differentially regulated proteins by using Gene Ontology (go). **(B)** Proteins involved in KEGG pathways, and showing a significant difference. **(C)** The network of significantly differentially expressed proteins was analyzed using String. The nodes represent proteins; the blue lines represent database evidence; pink lines represent experimental evidence; yellow lines represent mining evidence; black lines represent evidence of co-expression; and green lines represent neighborhood evidence.

Gene ontology (GO) analysis was used to assign functions to the differentially expressed proteins (DEPs) with respect to their identification as cellular components, molecular function, and involvement in biological processes (**Figure 2A**). The top three enriched GO terms under “biological process” were “metabolic process,” “cellular process,” and “single-organism process.” An analysis of the “cellular component” top five categories is presented in **Figure 2A**. “Cell” had the most DEPs, followed by “membrane” and “macromolecular complex.” For the proteins classified as those involved in molecular function, “catalytic activity,” “binding,” and “transporter activity” were the most prominent categories.

In addition, ribosomal proteins were analyzed in our study, because ribosomal proteins are the important part of a ribosome, and the ribosome can interact with azithromycin (Bahari et al., 2017). Azithromycin reversibly binds to the bacterial ribosomal subunits and inhibits the progression of nascent proteins by blocking their exit tunnel in bacterial protein biosynthesis (Dubravko and Roberto, 2016). **Table 3** shows that most of the ribosomal proteins were significantly up-regulated, especially the 50S ribosomal protein L33, 50S ribosomal protein L23, 50S ribosomal protein L19, 50S ribosomal protein L14, 30S ribosomal protein S14, 30S ribosomal protein S21, 30S ribosomal protein S9, and 30S ribosomal protein S2. In addition, the interactions showing significant difference were analyzed by the String analysis, which was a network constituted by protein-protein interactions. **Figure 2C** shows active interactions among the 30S ribosomal protein S21, 30S ribosomal protein S14, 50S ribosomal protein L23, 50S ribosomal protein L14, and 50S ribosomal protein L19. Ribosomal proteins involved in the protein translational machinery were impacted by azithromycin, which interfered with protein synthesis. The expression of about 148 proteins was altered: 58 showed a significant increase and 88 showed a significant decrease (**Supplementary Table S1**), which can be attributed to the change in ribosomal proteins.

Further analysis of the pathways affected by 1/2-MIC azithromycin was performed by KEGG pathway analysis, which provided the complex interactive link between multiple identified proteins for their commonly known networks and other cellular metabolic information. In this study, the differential proteins were analyzed; the pathways containing a minimum of three distinct proteins are shown in **Figure 2B**. Twenty pathways were significantly affected by azithromycin, including biosynthesis of amino acids, metabolism of glyoxylate and dicarboxylate, ABC transporters, metabolism of pyruvate and carbon, and histidine metabolism, among others. From our preliminary study, histidine biosynthesis pathway, an old and important bacterial metabolic pathway, played an important role in biofilm formation (Xu et al., 2017; Zhou et al., 2018), and IGPD, another important protein involved in histidine biosynthesis, could affect biofilm formation by *S. xylosus* (Chen et al., 2017; Zhou et al., 2018). Therefore, the histidine biosynthesis pathway was studied in detail. **Table 1** lists all proteins involved in histidine biosynthesis that showed down-regulation. In fact, IGPD showed a marked reduction. The proteins involved in histidine degradation into glutamate were also studied, and all of them were found to be reduced in the azithromycin-treated group. Histidinol dehydrogenase activity had also decreased significantly (**Table 2**).

**Table 1:** Changes in the expression of the proteins involved in histidine biosynthesis after azithromycin treatment.

Accession	Proteins	Fold change
A0A068E9J3^∗^	Imidazoleglycerol-phosphate dehydratase	0.42
A0A068E4P8^∗^	1-(5-phosphoribosyl)-5-[(5-phosphoribosylamino)methylideneamino] imidazole-4-carboxamide isomerase	0.52
A0A068E2C8^∗^	Histidinol dehydrogenase	0.65
A0A068E5N4	Histidine biosynthesis bifunctional protein HisIE	0.67
A0A060MIS4	Imidazole glycerol phosphate synthase subunit HisH	0.69
A0A060MPH2	ATP phosphoribosyltransferase	0.87
A0A068E8R1	Histidinol-phosphate aminotransferase	0.89

*^∗^P ≤ 0.05.*

**Table 2:** Changes in the expression of the proteins involved in the degradation of histidine into glutamate after azithromycin treatment.

Accession	Proteins	Fold change
A0A068E547^∗^	Formimidoylglutamase	0.43
A0A060MSD4	Histidine ammonia-lyase	0.76
A0A068E633	Urocanate hydratase	0.83
A0A068E2P9	Imidazolonepropionase	0.87

^∗^*P* ≤ 0.05.

### Histidine Content Determination and Real-Time PCR Analysis

As all proteins involved in histidine biosynthesis were down-regulated, we compared the change in the histidine content in the treated and untreated groups. Only a slight change was observed in the histidine content in the azithromycin-treated group (**Figure 5A**). The proteins involved in the degradation of histidine into glutamate were also down-regulated, which can help further interpret the mechanism involved in histidine biosynthesis, a point that will be investigated in the future.

iTRAQ analysis showed that IGPD and histidinol dehydrogenase involved in histidine biosynthesis and histidine degradation into glutamate were significantly down-regulated. Therefore, real-time PCR was performed to test *hisB* (IGPD) and *hutG* (histidinol dehydrogenase). The related genes *hisB* and *hutG* were selected (**Table 4**), and the results of gene expression analysis are presented in **Figure 5B**. The levels of *hisB* and *hutG* were significantly down-regulated (**Figure 5B**), which were consistent with the results of proteomic analysis.

### Molecular Docking Between IGPD and Azithromycin

**FIGURE 3:**
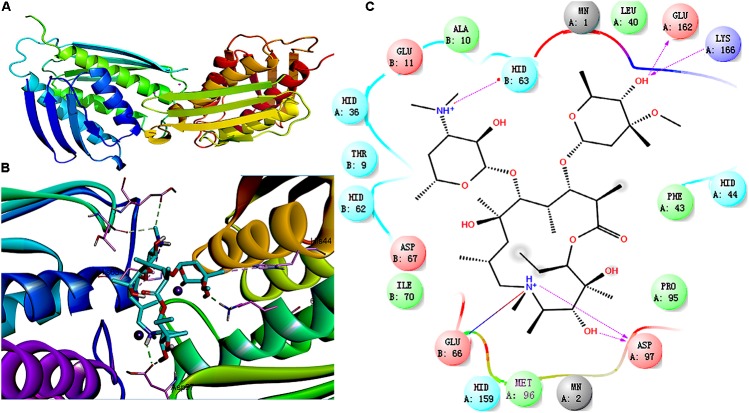
Molecular docking between IGPD and azithromycin. **(A)** Superposition of ligand-free dimers of IGPD, showing different flap conformations. **(B)** 3D-docked images of azithromycin with IGPD. **(C)** Binding interactions between IGPD and azithromycin. Purple lines, H-bond; red lines, salt bridge

Imidazole glycerophosphate dehydratase plays an important role in biofilm formation by *S. xylosus.* IGPD showed a sharp decline after treatment with 1/2-MIC azithromycin (0.25 μg/mL), and therefore, molecular docking was used to predict the relation between IGPD and azithromycin. As shown in **Figure 3**, about six bonds were noted: (1) two direct H-bonds were formed between Asp97 and C_3_-OH/NH^+^ of lactone, (2) a salt bridge was produced between Glu66 and NH^+^ of lactone, (3) two hydrogen bonds were generated between the hydroxyl of hexosamine and Glu162/Lys166, and (4) a H-bond was formed between Hid63 and NH^+^ of hexosamine. Azithromycin can be combined with IGPD. However, we also found that the active groups of three hydroxyl and seven oxygen atoms in azithromycin did not have any bond with IGPD. Thus, it is necessary to further study the interaction between azithromycin and IGPD.

### SPR Analysis

**FIGURE 4:**
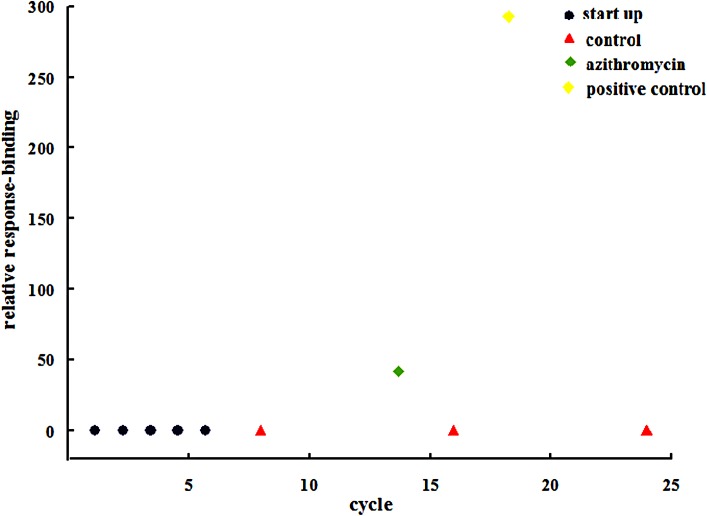
Surface plasmon resonance analysis of the bond between azithromycin and IGPD.

Surface plasmon resonance analysis was used to further evaluate the interplay between the drug and IGPD. **Figure 4** shows that the relative response binding of azithromycin is <50, and that the value for the positive control was much higher than that for azithromycin.

**FIGURE 5:**
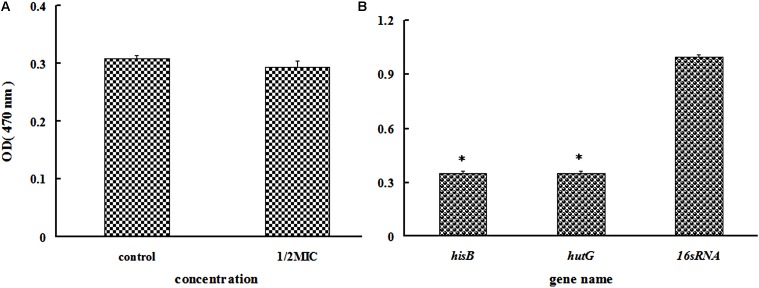
**(A)** Determination of the histidine content of *Staphylococcus xylosus* 700404 untreated or treated with azithromycin (1/2-MIC). **(B)** Effect of azithromycin (1/2-MIC) on the mRNA expression of *hisB* and *hutG* genes in *Staphylococcus xylosus* 700404. The expression was normalized to that of 16S rRNA. Controls refer to the absence of cefquinome. ^∗^*P* < 0.05 indicated significant difference, compared to the control bacteria. Data are expressed as the mean + SD.

## Discussion

In this study, the relationship between biofilm formation by *S. xylosus* and azithromycin was investigated. Sub-inhibitory concentrations of azithromycin significantly reduced (*P* < 0.05) biofilm formation, a finding consistent with those of previous studies (Gui et al., 2014; Yang et al., 2016).

To investigate the mechanisms underlying the effect of azithromycin, proteomic analysis was performed by the iTRAQ method and DEP studies. Compared to the control group, about 148 proteins showed significantly different expression in the *S. xylosus* treated with 1/2-MIC azithromycin (**Supplementary Table S1**). The interaction between azithromycin and ribosomes might also influence DEPs. Azithromycin can plug inside the ribosomal nascent peptide exit tunnel (NPET), which plays an active role in translational control (Marin, 2008). All nascent polypeptides must traverse and exit this tunnel, and the interactions of the nascent chain with the exit tunnel can modulate the rate of protein synthesis, leading to pausing or stalling of translational elongation, and affecting protein synthesis (Wilson and Beckmann, 2011). In addition, the expression of most ribosomal proteins increased after treatment with 1/2-MIC azithromycin (**Table 3**), which could be another reason causing alteration of protein expression. The ribosomal proteins are important components of the ribosome, the primary protein synthesis machine in the cell (Lamichhane et al., 2016). They play a key role in translation, most likely because of their significant functions that direct the folding and structure maintenance of the ribosome (Lu et al., 2015). Therefore, changes in the expression of ribosomal proteins can affect the ribosome, causing subsequent interruption or interference of protein synthesis, and alteration of protein expression can influence biofilm formation by *S. xylosus*, especially that of the proteins involved in histidine biosynthesis (Cabral et al., 2011; Chen et al., 2017).

**Table 3:** Changes in the ribosomal protein expression after azithromycin treatment.

Accession	Proteins	Fold change
A0A060MD61^∗^	50S ribosomal protein L33	1.51
A0A068E6D9^∗^	50S ribosomal protein L23	1.33
A0A068E5E8^∗^	50S ribosomal protein L19	1.32
A0A060MQI6^∗^	50S ribosomal protein L14	1.31
A0A068E7K6	50S ribosomal protein L25	1.29
A0A060MQ55	50S ribosomal protein L31 type B	1.28
A0A060MI35	50S ribosomal protein L13	1.28
A0A068EEL9	50S ribosomal protein L1	1.26
A0A060MQJ0	50S ribosomal protein L22	1.25
A0A060MD61	50S ribosomal protein L33	1.25
A0A068E7V6	50S ribosomal protein L10	1.25
A0A060MNZ8	50S ribosomal protein L4	1.24
A0A060MNX4	50S ribosomal protein L18	1.22
A0A060MGY5	50S ribosomal protein L21	1.21
A0A060MHU5	50S ribosomal protein L20	1.21
A0A060MQJ5	50S ribosomal protein L3	1.21
A0A060MI88	50S ribosomal protein L5	1.21
A0A060MQH5	50S ribosomal protein L6	1.21
A0A060MEP5	50S ribosomal protein L30	1.20
A0A060MES4	50S ribosomal protein L29	1.18
A0A060MMS2	50S ribosomal protein L28	1.17
A0A060MEP0	50S ribosomal protein L36	1.16
A0A060MJK7	50S ribosomal protein L11	1.12
A0A060MJ03	50S ribosomal protein L15	1.11
A0A060MI93	50S ribosomal protein L16	1.11
A0A060MDK6	50S ribosomal protein L35	1.09
A0A068E9H7	50S ribosomal protein L7/L12	1.09
A0A060MI39	50S ribosomal protein L17	1.08
A0A060MNZ2	50S ribosomal protein L24	1.08
A0A060MHS6	50S ribosomal protein L27	1.07
A0A060MCM6	50S ribosomal protein L33	1.02
A0A060MER3^∗^	30S ribosomal protein S14 type Z	1.69
A0A060MHF0^∗^	30S ribosomal protein S21	1.43
A0A060MEN2^∗^	30S ribosomal protein S9	1.30
A0A060MNZ5^∗^	30S ribosomal protein S3	1.30
A0A068E7K7	30S ribosomal protein S2	1.27
A0A060MAS8	30S ribosomal protein S12	1.25
A0A068E5R8	50S ribosomal protein L32	1.22
A0A060MES5	50S ribosomal protein L2	1.22
A0A060MDC8	30S ribosomal protein S20	1.21
A0A068EB99	30S ribosomal protein S4	1.20
A0A060MIZ6	30S ribosomal protein S13	1.19
A0A060MJ23	30S ribosomal protein S8	1.18
A0A060MI71	30S ribosomal protein S5	1.18
A0A060ME53	30S ribosomal protein S7	1.18
A0A060MLB4	30S ribosomal protein S16	1.15
A0A060MJ42	30S ribosomal protein S19	1.14
A0A060MJ47	30S ribosomal protein S10	1.11
A0A060MGR7	30S ribosomal protein S18	1.11
A0A060MQE3	30S ribosomal protein S11	1.10
C6ZDI5	30S ribosomal protein S1	1.02
C6ZDG4	30S ribosomal protein S6	0.94
A0A060MLE4	30S ribosomal protein S15	0.88
A0A060MFY5	30S ribosomal protein S14	0.84

**Table 4:** Primers used for real-time PCR in this study.

Name	Sequence (5′–3′)
*hisB*-F	TAACACTGCTGAAACACAACTATC
*hisB*-R	CTTCTGTATCACCATTTGCTTCG
*hutG*-F	TTTGATACAAGAGAGGCAGAAGG
*hutG*-R	TCCGCATAAACATAACCGATACC
16sRNA-F	CGGGCAATTTGTTTAGCA
16sRNA-R	ATTAGGTGGAGCAGGTCA

The histidine biosynthesis pathway is an important pathway found in bacteria, fungi, and plants (Pahwa et al., 2010). It comprises nine enzymatic reactions undertaken by seven proteins in the unbranched pathway (Nunes et al., 2011). In our study, all proteins participating in the histidine biosynthesis pathway were down-regulated. A change in the histidine biosynthesis pathway may impact nitrogen metabolism, which can further affect the growth and adhesion of bacteria, two parameters essential for biofilm formation (Bou et al., 2014). Changes to bacterial growth can affect cell density, which is further related to the quorum sensing system that regulates biofilm formation (Ding et al., 2017; Rajkumari et al., 2018). In addition, biofilm formation mainly includes three processes, and the adhesion of cells to surfaces is a key step (Yang et al., 2016). Therefore, down-regulation of the histidine biosynthesis pathway might be another reason for the inhibition of biofilm formation by *S. xylosus* by azithromycin.

Imidazole glycerophosphate dehydratase catalyzes the sixth step in the histidine biosynthesis pathway, and it has been identified as a potential herbicide target because of its important role in histidine biosynthesis (Ahangar et al., 2013; Bisson et al., 2015). In this study, IGPD had a 0.42-fold change after treatment with 1/2-MIC azithromycin, which might be the cause of reduced biofilm formation by *S. xylosus.* Because IGPD plays a very important role in biofilm formation by *S. xylosus*, the ability to form biofilms is severely affected in the *hisB* deletion mutant strain compared to the wild-type strain (Zhou et al., 2018). Further, our previous study found that as a potential target, IGPD could interact with drugs such as cefquinome, baicalin, fisetin, and ferulic acid (Chen et al., 2017). Therefore, we suspected that azithromycin interacted with IGPD, leading to the decline of IGPD, with the subsequent inhibition of biofilm formation by *S. xylosus*. To verify this hypothesis, a 3D structure of IGPD was constructed, and azithromycin was employed in the molecular docking study. Molecular docking is a method to identify the preferred orientation of a molecule in the active sites of a protein, and can predict the interactions between small molecules and the protein (Śledź and Caflisch, 2017). About six bonds were observed between azithromycin and IGPD, including five hydrogen bonds and one salt bridge (**Figure 3**). Therefore, we speculated that azithromycin can be combined with IGPD. However, we also found about 10 active atoms in azithromycin that did not interact with the biologically active site of IGPD. Therefore, SPR method was used to further test the relation between azithromycin and IGPD in our study. SPR method, as a label-free technique, can directly monitor specific drug and protein interactions, and it has been used to study drug-target interactions in recent years (Chen et al., 2010). We found that azithromycin hardly interacted with IGPD. Therefore, IGPD down-regulation was not caused because of the binding complex with azithromycin. It might have been caused by the significant decline in the *hisB* transcription level in the azithromycin-treated group, which was measured by real-time PCR.

Although all proteins with catalytic function in the histidine biosynthesis pathway were down-regulated in the azithromycin-treated group, histidine content showed slight change. To investigate this phenomenon, the pathway of histidine degradation into glutamate was also studied. This pathway is a very important histidine metabolic process, which enables the organisms to use histidine as a source of glutamate, and the glutamate can be used as a general source of carbon, nitrogen, and energy for growth (Magasanik et al., 1971). All proteins listed in **Table 2** showed down-regulated expression, especially formimidoylglutamase, which can catalyze the conversion of *N*-formimidoyl-L-glutamate to L-glutamate and formamide (Bender, 2012). Down-regulated expression of these proteins might reduce the consumption of histidine (**Supplementary Figure S1**), which can further result in similar histidine content in the treated and untreated groups, a phenomenon that needs to be studied further in the future.

Our findings showed that azithromycin can effectively inhibit biofilm formation by *S. xylosus* 700404 *in vitro.* The proteins involved in the histidine biosynthesis pathway were down-regulated on treatment with azithromycin (1/2-MIC), especially IGPD. This reduction in IGPD can be attributed to the regulation of histidine gene expression, rather than to the interplay between IGPD and azithromycin.

## Required Repositories

The proteome profiling data described in this manuscript is available in a public repository, as described in the guidelines.

## Author Contributions

YhL designed the experiments. WD undertook all experiments and wrote the manuscript. BG, XC, and YyL modified the manuscript. YZ and QQ took part in the TCP assay and SEM. WC, YY, and MC conducted the molecular docking experiments.

## Conflict of Interest Statement

The authors declare that the research was conducted in the absence of any commercial or financial relationships that could be construed as a potential conflict of interest.

## Supplementary Material

FIGURE S1 KEGG pathway map of histidine metabolism.

TABLE S1 List of proteins expressed by azithromycin-treated Staphylococcus xylosus, showing a significant difference.
