# Supplementary figures and images for: Azithromycin Inhibits Biofilm Formation by Staphylococcus xylosus and Affects Histidine Biosynthesis Pathway
Source: Front Pharmacol. 2018 Jul 10;9:740. doi: 10.3389/fphar.2018.00740 (PMC6048454; doi:10.3389/fphar.2018.00740)

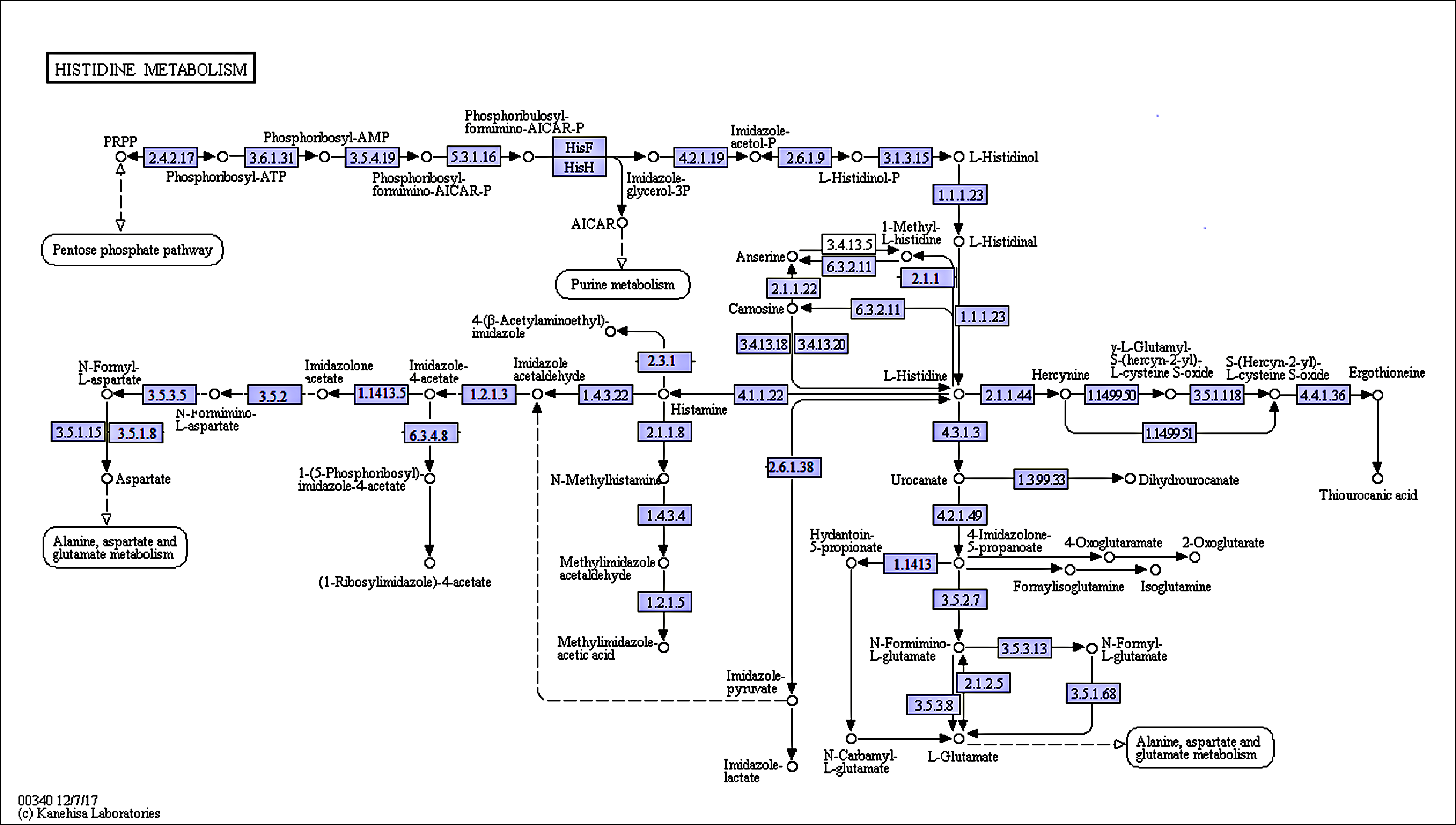

Supplement: FIGURE S1 — KEGG pathway map of histidine metabolism. [file Image_1.TIF]
