# Supplementary material for: Azithromycin Inhibits Biofilm Formation by Staphylococcus xylosus and Affects Histidine Biosynthesis Pathway
Source: Front Pharmacol. 2018 Jul 10;9:740. doi: 10.3389/fphar.2018.00740 (PMC6048454; doi:10.3389/fphar.2018.00740)
Supplement: TABLE S1 — List of proteins expressed by azithromycin-treated Staphylococcus xylosus, showing a significant difference. [file Table_1.doc]

**Supplementary Table:** List of proteins expressed by azithromycin-treated *Staphylococcus xylosus*, showing a [significant](javascript:void(0);) [difference](javascript:void(0);)

| Accession | Proteins | Fold change |
| --- | --- | --- |
| A0A068E269 | Uncharacterized protein | 5.49 |
| A0A068E9C1 | Poly-gamma-glutamate synthase subunit PgsB/CapB | 5.21 |
| A0A068E4Z9 | ABC transporter, ATP-binding protein | 2.36 |
| A0A068E690 | L-lactate dehydrogenase | 1.85 |
| K8DVZ1 | Uncharacterized protein | 1.80 |
| A0A060MLZ9 | Putative DUF1027 domain protein | 1.78 |
| A0A060MER3 | 30S ribosomal protein S14 type Z | 1.69 |
| A0A068E8G1 | DNA polymerase IV | 1.69 |
| A0A060MFV6 | Na(+) H(+) antiporter subunit F | 1.69 |
| Q6PY05 | Superoxide dismutase (Fragment) | 1.64 |
| A0A2G2R7F1 | N-acetylmuramoyl-L-alanine amidase | 1.62 |
| A0A068E5T2 | Putative permease of the major facilitator superfamily | 1.61 |
| A0A068EAX9 | Allantoate amidohydrolase | 1.53 |
| A0A068E9E8 | Aldehyde dehydrogenase A | 1.52 |
| A0A060MD61 | 50S ribosomal protein L33 | 1.51 |
| A0A068E5I4 | Acetylornithine deacetylase | 1.51 |
| A71Y64 | Probable transglycosylase isaA | 1.50 |
| A0A068E321 | Uncharacterized protein | 1.49 |
| A0A068E914 | Putative peptidase | 1.49 |
| A0A060MN37 | Glycerol-3-phosphate acyltransferase | 1.48 |
| A0A060MBV1 | UPF0413 protein | 1.44 |
| A0A060MHF0 | 30S ribosomal protein S21 | 1.43 |
| A0A068E5M8 | Zn(II) and Co(II) transmembrane diffusion facilitator | 1.42 |
| A0A068EA68 | Flavohemoprotein | 1.42 |
| A0A068EA51 | Integral membrane protein | 1.41 |
| A0A068ECN8 | ATP-dependent protease ATPase subunit HslU | 1.41 |
| A0A068E327 | Zinc ABC transporter, periplasmic-binding protein ZnuA | 1.40 |
| A0A068EE50 | Uncharacterized protein | 1.40 |
| A0A068E3G5 | Nitroreductase family protein | 1.39 |
| A0A068E8T2 | Uncharacterized protein | 1.37 |
| A0A060MK74 | Aldehyde dehydrogenase | 1.37 |
| A0A068E6Z9 | Diphosphomevalonate decarboxylase | 1.37 |
| A0A060MM27 | Exodeoxyribonuclease 7 small subunit | 1.36 |
| A0A068E3T7 | Transcriptional regulator, MarR family | 1.36 |
| A0A068E945 | Long-chain-fatty-acid--CoA ligase | 1.35 |
| A0A068EAW5 | Lipid A export ATP-binding/permease protein MsbA | 1.35 |
| A0A068E863 | Segregation and condensation protein B | 1.35 |
| A0A060MSF6 | Putative amino acid transporter | 1.35 |
| A0A068E2S9 | Putative monooxygenase | 1.35 |
| A0A068ECN8 | ATP-dependent protease ATPase subunit HslU | 1.34 |
| A0A060MMJ8 | D-aminoacyl-tRNA deacylase | 1.34 |
| A0A060MSC8 | Uncharacterized protein | 1.33 |
| A0A068E6D9 | 50S ribosomal protein L23 | 1.33 |
| A0A068E6N0 | Magnesium and cobalt efflux protein CorC | 1.33 |
| A0A068E5I6 | Putative preQ0 transporter | 1.33 |
| A0A068E6Q7 | Putative sugar transport protein | 1.33 |
| A0A068E538 | Putative glycosyl/glycerophosphate transferases involved in teichoic acid biosynthesis TagF/TagB/EpsJ/RodC | 1.32 |
| A0A060MQ60 | Fructose-bisphosphate aldolase | 1.32 |
| A0A068EAU2 | Putative O-methyltransferase | 1.32 |
| A0A068EB49 | SAM-dependent methyltransferase, MraW methylase family | 1.32 |
| A0A068E5E8 | 50S ribosomal protein L19 | 1.32 |
| A0A068E6P4 | Uncharacterized protein | 1.32 |
| A0A068E9H6 | Argininosuccinate synthase | 1.31 |
| A0A068E5D0 | N utilization substance protein B homolog | 1.31 |
| A0A068E497 | Putative exported protein | 1.31 |
| A0A068E4G1 | Pyruvate oxidase, CidC | 1.31 |
| A0A060MQI6 | 50S ribosomal protein L14 | 1.31 |
| A0A068E5R1 | Glycine betaine/carnitine/choline ABC transporter opuCC | 1.31 |
| A0A060MEN2 | 30S ribosomal protein S9 | 1.30 |
| A0A060MNZ5 | 30S ribosomal protein S3 | 1.30 |
| A0A068EDF8 | Ornithine aminotransferase | 0.65 |
| A0A068E3A1 | Pyrrolidone-carboxylate peptidase | 0.65 |
| A0A068E1P0 | Uncharacterized protein | 0.65 |
| A0A068E2C8 | Histidinol dehydrogenase | 0.65 |
| A0A068E6R2 | Cobalamin-independent methionine synthase II | 0.65 |
| A0A060MM81 | Putative phosphoesterase SXYL_01917 | 0.65 |
| A0A060MH80 | Phosphoenolpyruvate carboxykinase [ATP] | 0.64 |
| A0A060MBM8 | Glycine cleavage system H protein | 0.64 |
| A0A068E2W2 | N-succinyldiaminopimelate aminotransferase | 0.64 |
| A0A068E7D5 | Threonine synthase | 0.64 |
| A0A068E481 | Lipoprotein | 0.63 |
| A0A068E8C4 | Oligopeptide transport ATP-binding protein OppD | 0.63 |
| A0A068E5U9 | Homoserine dehydrogenase | 0.63 |
| A0A2G2RCD7 | Proline dehydrogenase | 0.62 |
| A0A068E3N8 | Uncharacterized protein | 0.62 |
| A0A068E606 | Teicoplanin resistance associated membrane protein TcaB | 0.62 |
| A0A068E6N5 | Uncharacterized protein | 0.62 |
| A0A060MLT7 | Preprotein translocase subunit SecG | 0.62 |
| A0A068E8G3 | Putative membrane protein YeiH | 0.62 |
| A0A068EAB9 | Glucosamine-6-phosphate deaminase | 0.62 |
| A0A068E4D6 | Uncharacterized protein | 0.62 |
| A0A068E6T0 | Oligopeptide transport system permease protein OppC | 0.62 |
| A0A068E874 | Chorismate synthase | 0.61 |
| A0A068E8Z3 | Manganese ABC transporter, periplasmic-binding protein SitA | 0.61 |
| A0A068E669 | Bifunctional autolysin Atl | 0.61 |
| A0A068E8B7 | Uncharacterized protein | 0.61 |
| A0A068E6U0 | Glucose-6-phosphate isomerase | 0.61 |
| A0A060MBN2 | Lipoprotein | 0.60 |
| A0A068E3T6 | FmtB protein | 0.60 |
| A0A068E526 | Gamma-aminobutyrate:alpha-ketoglutarate aminotransferase | 0.60 |
| A0A068E4N6 | Beta-N-acetylhexosaminidase | 0.59 |
| A0A068ED26 | Heme A synthase | 0.59 |
| A0A060MH08 | Glyceraldehyde-3-phosphate dehydrogenase | 0.59 |
| A0A2G2R7V4 | Uncharacterized protein | 0.59 |
| A0A068E243 | Lactonase Drp35 | 0.58 |
| A0A068EE66 | Inosose isomerase | 0.58 |
| A0A2G2R961 | Outer surface protein | 0.57 |
| A0A068ECI5 | Glycerol uptake facilitator protein | 0.57 |
| A0A068EF52 | ABC transporter ATP-binding protein | 0.57 |
| A0A068E2Y3 | Putative permease of the major facilitator superfamily | 0.57 |
| A0A068E9J8 | D-alanine--poly(phosphoribitol) ligase subunit 1 | 0.57 |
| A0A068EB44 | 6,7-dimethyl-8-ribityllumazine synthase | 0.56 |
| A0A060MRD2 | 1-pyrroline-5-carboxylate dehydrogenase | 0.56 |
| A0A068E8Q9 | Ribonucleotide reductase of class III (Anaerobic), large subunit | 0.56 |
| A0A068E8J7 | Oxidoreductase, short chain dehydrogenase/reductase family | 0.55 |
| A0A068E340 | Alpha-acetolactate decarboxylase | 0.55 |
| A0A060MLC9 | Uncharacterized protein | 0.55 |
| A0A068E8D8 | Putative membrane protein | 0.53 |
| K8DVF8 | Putative regulator of sorbitol operon | 0.53 |
| A0A068E514 | Pyruvate phosphate dikinase | 0.53 |
| A0A068E204 | Uncharacterized protein | 0.53 |
| A0A068E499 | Oligopeptide ABC transporter, periplasmic oligopeptide-binding protein OppA | 0.53 |
| A0A068E2L5 | Glycerate kinase | 0.53 |
| A0A068E7D1 | Catalase | 0.52 |
| A0A068E1Q3 | Putative pyruvate, phosphate dikinase regulatory protein | 0.52 |
| A0A068E4P8 | 1-(5-phosphoribosyl)-5-[(5-phosphoribosylamino)methylideneamino] imidazole-4-carboxamide isomerase | 0.52 |
| A0A068E6J5 | Uncharacterized protein | 0.51 |
| A0A068E433 | Universal stress protein family | 0.51 |
| A0A060MCL3 | Uncharacterized protein | 0.51 |
| A0A060MJN5 | 3-oxoacyl-[acyl-carrier protein] reductase | 0.51 |
| A0A068EDD0 | Processive diacylglycerol beta-glucosyltransferase | 0.51 |
| A0A060MK38 | LysR family transcriptional regulator | 0.50 |
| A0A068E9C9 | 2-dehydro-3-deoxyphosphogluconate aldolase | 0.50 |
| A0A068E4J3 | Putative sialic acid transporter | 0.50 |
| A0A068E2S8 | L-lactate dehydrogenase | 0.49 |
| A0A068E9F7 | Sodium/proline symporter | 0.49 |
| A0A060MB56 | Na(+) H(+) antiporter subunit E | 0.49 |
| A0A068E407 | Acetyl-coenzyme A synthetase | 0.49 |
| A0A060MF11 | Oligopeptide transport system permease protein OppB | 0.47 |
| A0A068E4G9 | Acetylornithine deacetylase | 0.47 |
| A0A068EF38 | Alpha-galactosidase | 0.46 |
| A0A060MNG4 | Nucleoside diphosphate kinase | 0.45 |
| A0A068E1R4 | Succinate-semialdehyde dehydrogenase [NADP+] | 0.45 |
| A0A068E3A0 | Arginase | 0.45 |
| A0A068E547 | Formimidoylglutamase | 0.43 |
| A7IY64 | Probable transglycosylase IsaA | 0.43 |
| A0A068E8W0 | Circadian phase modifier, NCAIR mutase (PurE)-related protein | 0.42 |
| A0A068E9J3 | Imidazoleglycerol-phosphate dehydratase | 0.42 |
| A0A068E7B7 | Uncharacterized protein | 0.41 |
| A0A068E533 | Tautomerase | 0.40 |
| A0A068E2L0 | UPF0272 protein SXYLSMQ121_0200 | 0.38 |
| A0A068EER8 | Transcription-repair-coupling factor | 0.32 |
| A0A068E6R7 | Sodium/alanine symporter family protein | 0.30 |
| A0A068E843 | Hydrolase (HAD superfamily) | 0.28 |
| A0A068E3Z3 | PhnB protein | 0.27 |
| A0A068E276 | Uncharacterized protein | 0.25 |
| A0A068E8L1 | Glutamine synthetase | 0.23 |
| A0A068E9S0 | Uncharacterized protein | 0.12 |
